# Talking with Each Other about Science

**DOI:** 10.1002/hast.4978

**Published:** 2025-04-17

**Authors:** Dena S. Davis

**Keywords:** Francis Collins, NIH, evangelical Protestantism, Human Genome Project, public trust in science, bioethics

## Abstract

The Road to Wisdom: On Truth, Science, Faith, and Trust, *the optimistic title of Francis S. Collins's new book, hides a realistic appraisal of our current polarized country. Collins tries to tackle, in everyday language, why people are having such a hard time figuring out what is truth, how to think about science, how religious faith fits into this, and how and whom we can trust. “Misinformation, disinformation, fear … are constantly trying to knock us into the ditch,” he observes. His prescriptions are personalized and actionable—for example, talking to a neighbor whose views are different from yours*.

## Book Review

The Road to Wisdom: On Truth, Science, Faith, and Trust, By Francis S. Collins . Little, Brown and Company. 2024.

In 2009, when Francis S. Collins was appointed to head the National Institutes of Health, after serving as head of the National Human Genome Research Institute (where, for about nine months in 1998 to 1999, I worked with him as a visiting professor), the *New York Times* described the scientific community as not entirely enthusiastic about him. Some disagreed with his commitment to “big science,” especially the Human Genome Project. Others, however, were skeptical about Collins's very public Christian evangelical faith, highlighted in books and talks. Genetics and evangelicalism were often considered to be a fraught and inherently hostile combination. It is true that a great deal of the criticism of genetic science came from Christian evangelicals, but I believed that that was all the more reason for nonevangelicals, and perhaps especially those with no religious affiliation, to applaud the selection of Collins as a brilliant choice. In his recent book, Collins states, “I have never encountered a situation where I found my scientific and spiritual worldviews to be in serious conflict” (p. 8). According to a 2023‐2024 Pew Research Center survey on religion, 26 percent of Americans label themselves as Christian evangelicals, making it the largest single religious group in American society. Surely, seeing someone who is both a respected scientist and a solid Christian should prove reassuring, or at least thought‐provoking, to the Christian who is trying to make sense of things.

In *The Road to Wisdom: On Truth, Science, Faith, and Trust*, Collins tries to tackle, in everyday language, why people are having such a hard time figuring out what is truth, how to think about science, how religious faith fits into this, and how and whom we can trust. “Misinformation, disinformation, fear … are constantly trying to knock us into the ditch” when it comes to a societal agreement about what is true and reliable, he observes (p. 210).

Often, of course, misinformation, disinformation, and fear are rooted in our country's ever‐worsening political polarization, and Collins's insights into talking across divides is particularly illuminating. As an academic who lives on the Upper West Side of New York City, I honestly do not know a single person who voted for the current U.S. presidential administration or who has not been vaccinated for Covid. Where I used to live, in semirural Pennsylvania, there was more of a mix. As we learn from sociologists, the country is self‐segregating into red and blue bubbles, and it is harder and harder to talk (civilly) with people one does not agree with. Collins tells us how to do this, in ways that are truly compelling.

One of the groups Collins has been involved with is Braver Angels, an organization founded in 2016 to help people who disagree on important topics converse civilly with one another. As Collins's Braver Angels friend Wilk Wilkinson says, “Things just aren't as bad as they would have you believe…. Shut off the news and talk to your neighbor” (p. 23). What Collins realized when he and Wilkinson faced each other in a 2023 public debate about science and the government and Covid is that Wilkinson had some good points to make about government policy that treats urban and rural areas without sensitivity to their differences. “With almost no early cases of serious illness happening in their mostly rural community, businesses and schools were still required to close,” Collins acknowledges. “Where was the consideration about the need for adapting recommendations to specific situations in this sprawling country?” (p. 16). Collins also realized that he and his science colleagues had not been very clear in explaining to people that scientists themselves were often uncertain about the best way of responding to this “novel” virus. That advice from the “experts” changed didn't mean that they were stupid or trying to fool people; it meant merely what the scientific process always means: that often you need to get it wrong before you can get it right.

As someone who taught a popular undergraduate course on Covid and ethics for three semesters, I was especially engaged with the Covid material in Collins's book. A very good explanation, easy to read and full of good facts, explains why the Great Barrington Declaration would not have worked. Signed by many infectious disease epidemiologists and public health scientists from around the world, this declaration suggested that we go about our business, “sequestering” only the elderly and letting herd immunity burn itself out. Collins shows why this approach would have caused the “unnecessary deaths” of tens of thousands or even hundreds of thousands of people (p. 110). A clear explanation of how Sweden, which adopted herd immunity, had many greater deaths than did its Scandinavian neighbors brings that point home (p. 112).

Collins's warmth can capture people's hearts when he speaks on PBS or in other settings, yet he can also be irritatingly sunny and optimistic—as he is at times in this book. I remember years ago, when Collins was trying to persuade people to get behind the 2008 Genetic Information Nondiscrimination Act, his naïve assumption that, once the act passed, patients would have no reason to believe that genetic discrimination would still exist—as if telling an African American person that, because the Fair Housing Act was passed in 1968, they should have no current concern about racial discrimination in housing. In *The Road to Wisdom*, his assertion that people who volunteer for the Peace Corps or Habitat for Humanity are “truly noble” (p. 139) feels overblown—ignoring any self‐serving interests they might have. Saying “There is nothing more un‐American than hating fellow Americans” (p. 22) suggests a poor exposure to history.

At the same time, his characterization of his own group, the American white evangelic church, is not a pretty picture. As both a scientist and someone who had worked and prayed for the Covid vaccine, Collins hates that white evangelicals are the group in this nation most likely to refuse the vaccine, and often for ludicrous reasons, like a fear that Bill Gates is putting personal tracking chips in the vaccines.

In talking about trust, Collins engagingly brings us along on his journey as a doctor needing medical care, but he also details the terrible experience of being hoodwinked by a graduate student who was falsifying data. Discussing how he relied on a doctor colleague to perform neck surgery to address a pain in his arm, Collins adds to the many powerful stories of physicians who are forced to accept the role of patient, of becoming the one who must trust rather than the one who is trusted. He also talks about the case, now spotlighted in the news once again, of Andrew Wakefield's fraudulent and self‐serving 1998 report, in the *Lancet*, that childhood vaccines were capable of causing autism. Although Wakefield's “research” was eventually thoroughly debunked and the paper retracted, a great deal of harm had been done, and the false research continues to drive some parents away from life‐saving vaccines. (What Collins does not discuss—what I have never seen discussed anywhere—is the responsibility that the *Lancet* shares in this debacle. Did the editors not see that there was something fishy in a report of only a dozen cases? Was it that difficult to discover that Wakefield had received monetary payments for his “scientific” report?)

It is when Collins is being most dire that he brings us back again to the solution. A true evangelical, he does finally believe that individuals can move mountains. How can we learn to trust others and find the right people to trust? Tribal affinity, he suggests, can be misleading, as can celebrities. Maybe you and your tribe are all deeply concerned about climate change; you experience feelings of kinship when you are with them. But should brotherhood about climate change also suggest that you trust people most like you on information about vaccines or nutrition? No, Collins suggests: you might do better to reach outside your tribe for other sources. This certainly brings to mind Linus Pauling, who won Nobel prizes in chemistry and peace in 1954 and 1962, respectively, and was beloved of leftists for his championship of nuclear disarmament, but whose quixotic support of vitamin C had little scientific support. Nevertheless, I am sure that Collins—himself a singer and guitar player—was very appreciative of Dolly Parton for getting her first Covid shot in public while singing to her tribe, “Vaccine, vaccine, vaccine, vaccine. I'm begging of you, please don't hesitate.”

Ultimately, says Collins, “[T]he real hope for the future rests with each of us; the road to wisdom runs right through our hearts and minds” (p. 216). He finds truth in these words of Wendell Berry: “If we are serious about these big problems, we have got to see that the solutions begin and end with ourselves” (qtd. on p. 211).

